# Validation of Mini-Mental Adjustment to Cancer scale in a Moroccan sample of breast cancer women

**DOI:** 10.1186/s12885-021-08755-y

**Published:** 2021-09-20

**Authors:** Mohammed El Amine Ragala, Jaouad El Hilaly, Lamiae Amaadour, Majid Omari, Achraf E. L. AsriI, Mariam Atassi, Zineb Benbrahim, Nawfel Mellas, Karima E. L. Rhazi, Karima Halim, Btissame Zarrouq

**Affiliations:** 1 Laboratory of Natural Substances, Pharmacology, Environment, Modeling, Health & Quality of Life, Faculty of Sciences Dhar El Mahraz, Sidi Mohamed Ben Abdellah University, P. B 1796 Atlas, 30003 Fez, Morocco; 2 Teachers Training College (Ecole Normale Superieure), Department of Biology and Geology, Sidi Mohamed Ben Abdellah University, P. B 5206 Bensouda, 30030 Fez, Morocco; 3 Laboratory of Pedagogical and Didactic Engineering of Sciences and Mathematics, Regional Center of Education and Training (CRMEF) of Fez. Rue Koweit, P.B 49 Agdal, 30050 Fes, Morocco; 4 R.N.E Laboratory, Multidisciplinary Faculty of Taza, Sidi Mohamed Ben Abdellah University, P. B 1223, Route Oujda, 35000 Fez, Morocco; 5 Medical Oncology Department, Hassan II University Hospital, Route Sidi Harazem, 30070 Fez, Morocco; 6 Laboratory of Epidemiology and Research in Health Sciences, Faculty of Medicine and Pharmacy, Sidi Mohamed Ben Abdellah University, 2.200 Route Sidi Harazem, 30070 Fez, KM Morocco; 7 Laboratory of Epidemiology, Clinical Research, and Public Health, Faculty of Medicine and Pharmacy, Mohamed I University, BP724 Hay Al Quods, 60000 Oujda, Morocco; 8 Mohamed VI University Hospital, Oujda, Morocco; 9 Teachers Training College (Ecole Normale Superieure), Department of Human and Social Sciences - Education Sciences, Sidi Mohamed Ben Abdellah University, P. B 5206 Bensouda, 30030 Fez, Morocco

**Keywords:** Adjustment, Cancer, Mini-MAC, Oncology, Psychometric, Validity

## Abstract

**Background:**

The Mini-Mental Adjustment to Cancer Scale (Mini-MAC) instrument is commonly used worldwide by professionals of oncology, but the scale has not, up to date, been validated in Arabic and Moroccan context, and there is an absence of data in the Moroccan population. This study aims to validate the Mini-MAC, translated and adapted to the Arabic language and Moroccan culture, in women with breast cancer.

**Methods:**

Data were analyzed in two successive phases. First, exploratory factor analysis (EFA) was used to assess the factor structure in the pilot sample (*N* = 158). Then, this structure was confirmed in the validation sample (*N* = 203) using confirmatory factor analysis (CFA).

**Results:**

Confirmatory factor analysis confirmed Watson’s original structure underlying the Mini-MAC items: Helpless/Hopeless, Anxious Preoccupation, Fighting Spirit, Cognitive Avoidance, and Fatalism. Absolute, incremental, and parsimonious fit indices showed a highly significant level of acceptance confirming a good performance of the measurement model. The instrument showed sufficient reliability and convergent validity demonstrated by acceptable values of composite reliability (CR =0.93–0.97), and average variance extracted (AVE = 0.66–0.93), respectively. The square roots of AVE were higher than factor-factor pairs correlations, and the Heterotrait-Monotrait ratio of correlations values were lesser than 0.85, indicating acceptable discriminant validity.

**Conclusions:**

reliability; and both convergent and discriminant validity tests indicated that the Arabic version of the Mini-MAC had a good performance and may serve as a valid tool measuring psychological responses to cancer diagnosis and treatment.

## Background

Nowadays, Non-communicable diseases (NCDs) represent the major health challenges for developing countries where the annual mortality rate exceeds largely that of all other diseases combined. Nearly 80% of NCDs deaths occur in low and middle-income countries [1]. As one of the African middle-income countries, Morocco is experiencing a substantial increase in the burden of NCDs along with a higher mortality rate, which represents around 75% of the total death in the country [2].

Breast cancer is one of the most common cancers in women around the world [3], the most prevalent deadly disease of women in low and middle-income countries, and is pictured as a symbol of the end of life [4]. It primarily concerns young women by targeting the breast, which is deemed as the most valuable thing in their life. The breast mirrors feminine valuableness, esthetic appearance, and motherhood [5].

Breast cancer is a leading cancer with an incidence of 10,136 new cases diagnosed in 2018, representing 19.2% of the overall cancer incidence and 36.9% of cancers diagnosed in Moroccan women. In terms of mortality, it is the second most common cancer for women, after lung cancer, with approximately 3518 deaths (12.3% of total cancer deaths) [6]. As the magnitude of the breast cancer epidemic continues to expand, healthcare interventions become cost-effective for the health system. Aiming to maintain and deliver an effective and affordable service for sick people, the Moroccan health system is embracing a package of measures in parallel with an emerging paradigm called the biopsychosocial model [7]. The latter seeks to integrate the psychological and the social dimensions in the traditional biomedical model. Psychological aspects are described in terms of cognitions, emotions, and behaviors, whereas social aspects consist of social norms of behavior, pressures to change behavior, social values on health, social status, and ethnicity [8]. Understanding these elements helps to determine the styles of psychological adjustments in cancer patients. Oncology specialists believe that coping style is very relevant in making decisions about adjuvant therapy or active follow-up, coping with side effects of treatment, and anxiety caused by uncertainty in the prognosis [9].

Coping and mental adaptation are the most widely studied concepts in psychosocial oncology [10]. Coping requires constantly changing cognitive and behavioral efforts to manage specific external or internal demands that are appraised as taxing or exceeding the resources of a person [11]. Greer et al. (1989) defined mental adjustment as cognitive and behavioral responses made by an individual to the diagnosis of cancer [12]. There are many scales to assess coping strategies such as “The Ways of Coping Checklist” (WCC) [13], “The Coping Inventory For Stressful Situations” (CISS), [14], and “The Coping with Health Injuries and Problems Scale” (CHIPS) [15]. On the other hand, some scales were developed to measure psychological adjustment like “The adjustment inventory” (adult form), which provides five measures of personal and social adjustment [16], “The ATT39 scale” used as a norm-referenced measure of emotional adjustment in diabetic patients [17], and “The Mental Adjustment to Cancer (MAC) Scale [12, 18].

The “Mental Adjustment to Cancer” (MAC) is a 40-item scale distributed over five subscales and has become a widely used instrument for assessing psychological adjustment in cancer patients. Several studies have tried to validate the MAC subscales, but they were not able to replicate the original factor structure [19, 20]. Hence, the original version was revised by Watson [21], leading to a 29-item scale with psychometric properties comparable to the original MAC scale. The shortened version was called the “Mini-Mental Adjustment to Cancer Scale” (Mini-MAC). It consists of five scales ((Helpless/Hopeless (HH), Anxious Preoccupation (AP), Fighting Spirit (FS), Cognitive Avoidance (CA), and Fatalism (FA).

The Mini-MAC, distilled from the MAC, has been translated into several other languages and investigated by many studies. Some authors have confirmed the original five-factor structure of the original Mini-MAC [22–25], while others proposed different structures of two [26], three [27], four [28–30], or five factors [22, 24, 25, 27, 31].

Among the five original factors, the fatalism subscale has sparked much debate among authors. Originally defined as a maladjustment style, fatalism was supposed to be adopted by patients to accept the situation as inevitable. Meanwhile, other studies [27] have considered it as an adaptive measure to religion, faith, reassessment, positive thinking, and acceptance [25, 30, 32]. Fatalism was found to be positively correlated with spirituality and active participation in religious practice but was not associated with a perceived lack of control and acceptance of results. The fatalism of Mini-MAC may be more associated with feelings of personal control and that it has a positive effect on the health of women with breast cancer [27].

Literature review reports incongruity and dissonance between authors’ findings regarding the Mini-MAC’s factor structure. This is due to many variables such as methodological issues, types and stages of cancer, and sample sizes. Together, these minor and major discrepancies of unstandardized studies would have impeded obtaining unified and robust factor solutions [33]. In addition, most validation studies of the Mini-MAC scale are not well-grounded and should be taken with caution. Indeed, though EFA is discouraged to draw substantive conclusions from a scale structure [34], few studies have conducted CFA to investigate the validation of the Mini-MAC scale [24, 28, 35].

Cancer is one of the most stressful diseases. Coping with cancer is a long-term process in which patients use different strategies to cope with the physical, psychological, and social controversies caused by the disease. The choice of coping strategies depends both on individual variability and temporal variability. The former arises from the patient’s personality traits, disease type, treatment, perceived support, etc. while the latter is associated with the stage of the disease and its course [36]. Though some strategies are in general better than others, most often they are associated with an optimal degree of psychological adjustment [37]. The provision of adequate mental support, which would enable patients to adopt constructive strategies to cope with the stress associated with the disease at each stage, is of great importance in the process of treatment and rehabilitation of patients with the cancer disease [36]. Since the psychological state of the patient can predict the progression and course of the disease [38], knowledge regarding adaptation to neoplastic disease should facilitate a detailed diagnosis and allow assessment of the mental, emotional and social status of a patient throughout the follow-up. Subsequently, appropriate intervention measures can be taken and the quality of life of cancer patients can be improved [36]. The MAC scale in its original version was used as a measure of psychological adjustment in cancer-diagnosed patients. Then, by the same token, the scale served various purposes: (a) to measure the clinical progress of patients and improve the clinical support provided to them; (b) to assess the effectiveness of psychotherapy; (c) to determine the effectiveness of various strategies and their evolution over time, and (d) to assess the possible impact of the adjustment on quality of life and survival [39]. As the Mini-MAC is strongly linked to the original scale (MAC) [21], we presume that the Arabic version of Mini-MAC would be of paramount usefulness for clinicians. It may help them to explore the four dimensions cited above, to permit the clinical understanding of the adaptation process affecting the quality of life and physical outcomes, and to link it prospectively to specific psychosocial services for early intervention in patients’ psychiatric morbidity.

The Mini-MAC instrument is commonly used worldwide by professionals of oncology, but the scale has not, up to date, been validated in Arabic and Moroccan context, and still, there is a lack of data on the Moroccan population. To this end, the present study aims to examine the factor structure, reliability, and validity of the Mini-MAC among breast cancer women in Morocco. In this line, the five first-order latent factors of the Mini-MAC original version were examined using Confirmatory factor analysis (CFA).

## Methods

### Mini-MAC utility

Some investigations have evaluated the feasibility of the Mini-MAC in research and clinical settings [40]. For research, the Mini-Mac is considered one of many tools used to evaluate the prognosis of patients with psychological disorders in longitudinal studies. To understand how psychological coping variables change as the duration of the cancer disease increases, the assessment is realized from diagnosis to follow-up [40]. In clinical care, the Mini-MAC can be used to measure adaptive and maladaptive coping of cancer patients that may be subjected to positive-coping programs [28]. Overall, the Arabic version of Mini-MAC may be useful both in psycho-oncology research and clinical practice. It can also assess the response to cancer diagnosis and treatments and categorize patients who may endure later psychological adjustment difficulties.

### Mini-MAC scale translation

The original version of the Mini-MAC scale was translated from English to Arabic, then reviewed by an expert group, and finally translated back into English by two independent translators who are unfamiliar with the Mini-MAC scale. Back translation was checked by English experts and corrected based on comments. After it was estimated as satisfying, the committee decided on the final Arabic version. The latter was then pilot tested asking 20 breast cancer women to complete and comment on the questionnaire. No item was noticed to be difficult to understand or confusing. Hence, no revision was made after the pilot test.

### Participants and procedure

A consecutive series of breast cancer women attending routine follow-up appointments at a public oncology hospital in Fez city were recruited. All the recruited women were under active treatment. They were included based on inclusion criteria: diagnosed with histologically confirmed breast cancer, aged 18 years or above. Of the total, 243 (67.3%) of the women were illiterate but understand spoken Arabic, and only 118 (32.7%) were able to read and write the language. The participants were able to consent, communicate, and carry out the interview. They were not confined to their chairs or beds and were aware of their cancer diagnosis*.* To measure the coping styles of patients with cancer, the recruited women were interviewed to fill the Mini-MAC questionnaire, and their demographic and clinical characteristics were collected. All the participants were notified about the aim of the study; their written approval to answer the questionnaires was given and approved by the hospital-university ethics committee of Sidi Mohamed Ben Abdellah University (N^°^ 24/18).

### Measures

Mini-Mental Adjustment to Cancer Scale (Mini-MAC) consists of 29 items on a four-point Likert scale ranging from “Definitely does not apply to me” to “Definitely apply to me”. It assesses five cognitive subscales: helpless/hopeless (HH, 8 items), anxious preoccupation (AP, 8 items), fighting spirit (FS, 4 items), cognitive avoidance (CA, 4 items), and fatalism (FA, 5 items). In phase 1, the 29-item Mini-MAC (original version) was piloted with 158 breast cancer women between February 2018 and April 2018. In phase 2, the modified 24-item Mini-MAC instrument (version 2) was distributed to 203 breast cancer women between Mai 2018 and July 2018.

### Statistical analyses

Statistical data analyses were performed on the R program with packages “psych”, “semTools”, and “lavaan”. Mini -MAC items on the whole sample were first analyzed by descriptive statistics. Then, the structure and internal consistency of the Mini-MAC questionnaire were tested. The suitability of the correlation matrix was verified, to ensure that it is factorized based on the Kaiser–Meyer–Olkin (KMO) test and Bartlett’s sphericity test [41]. The factorial structure of the Mini-MAC instrument was examined on the first sample (*N* = 158) using Exploratory Factor Analysis (EFA). The violation of the assumption of multivariate normality was assessed by Mardia’s test [42]. To determine the appropriate number of factors to extract, and due to the skewed ordinal, parallel analysis of polychoric correlations with PCA as a method of extraction was performed [43]. Whereas, the EFA was done by principal axis factoring (PAF), an extraction method, and oblimin rotation. To get the most parsimonious factor structure, the items with low communalities (less than 0.20), the significant cross-loaded items, and the unrepresentative ones were eliminated from the analysis. This was performed in a stepwise fashion; the EFA was rerun after each step [44]. The reliability of the Mini-MAC Scale was assessed based on its internal consistency, by determining Cronbach’s alpha coefficient. The theoretical model of the Mini-MAC instrument was tested by confirmatory factor analysis (CFA). A 24-item confirmatory factor analysis was conducted on the whole sample (*N* = 203) using item-level ordered categorical data because items’ level of measurement is ordinal. Hence, CFA was performed using a polychoric correlation matrix and diagonal weighted least squares (DWLS) robust estimation technique. The internal consistency was estimated by computing composite reliability (CR), convergent validity was assessed using the average variance extracted (AVE), and discriminant validity was tested by the Fornell-Larcker criterion and Hetereotrait-Monotrait (HTMT) ratio [45]. The Fitness of the measurement model was reported from three categories of incremental fit (CFI, IFI, AGFI); 2) absolute fit (RMSEA, GFI), and 3) parsimonious fit (Chisq/df).

## Results

### Sample characteristics

The study population consisted of two samples of patients with breast cancer. The first sample (N = 158) was analyzed by exploratory factor analysis, while the second one (*N* = 203) was tested by confirmatory factor analysis. The two samples presented similar demographic characteristics (Table 1). The mean age was 49.01 ± 11.38 (range 27–83) and 48.86 ± 11.65 (range 26–88) for the first and second samples, respectively. 68.35% of the patients in the first sample were married against 67.98% in the second. In terms of the level of education, most patients of the two samples were illiterate (67.09% versus 67.49%). Most of the patients lived in an urban environment, and stage II cancer dominated in the two samples (50.63% versus 50.25%).

**Table 1:** Socio-demographic and clinical characteristics of the participants

	Phase 1 (***N*** = 158)		Phase 2 (***N*** = 203)	
	Mean	N (%)	Mean	N (%)
*Age*^a^	49.01 ± 11.38 (Range 27–83)		48.86 ± 11.65 (Range 26–88)	
*Marital status*				
Unmarried		21 (13.29)		28 (13.79)
Married		108 (68.35)		138 (67.98)
Widowed		20 (12.66)		26 (12.81)
Divorced		9 (5.70)		11 (5.42)
*Employment*				
Employed		14 (8.86)		19 (9.36)
Unemployed		25 (15.82)		35 (17.24)
Housewife		96 (60.76)		121 (59.61)
Retiree		23 (14.56)		28 (13.79)
*Education*				
Illiterate		106 (67.09)		137 (67.49)
Primary education		29 (18.35)		39 (19.21)
Secondary education		17 (10.76)		19 (9.36)
Higher Education		6 (3.80)		8 (3.94)
*Living environment*				
Urban		90 (56.96)		119 (58.62)
Rural		68 (43.04)		84 (41.38)
*Cancer stage*^b^				
II		80 (50.63)		102 (50.25)
III		30 (18.99)		38 (18.72)
IV		48 (30.38)		59 (29.06)

^a^ (Mean ± SD), ^b^ Four missing values for *N* = 203.

### Exploratory factor analysis

The underlying factor structure of the Mini-MAC was examined by analyzing the data from the first convenience sample drawn from that part of the population that was easy to reach, which satisfied the inclusion criteria (*n* = 158). The sampling adequacy for performing the analysis was verified through the Kaiser–Meyer–Olkin test (KMO). The total KMO value was 0.89, and all KMO values for individual items were higher than 0.67, well above the acceptable limit of 0.60 [46]. Bartlett’s test of sphericity (χ2 = 3188.57, df = 406, *p* < .001) indicated that inter-item correlations were sufficiently large to perform EFA.

Parallel analysis of polychoric correlation with PCA as a method of extraction and Velicer MAP criterium supported the adequacy of a five-factor solution. Factorial analysis with Principal axis factoring (PAF) as extraction method and oblimin rotation has yielded the first structure resembling nearly the authentic Mini-MAC version (Table 2). A loading cutoff point of at least 0.30 was initially used. Items that failed to load higher than this threshold or loaded significantly onto multiple factors were rejected from all factors. After each run, the analysis of the rotated factor matrix showed the significant factor loadings and the changes in communalities values. If an observed variable was not significant, it was eliminated from the measurement model. In each case that a variable was dropped, the model was respecified and run again. This was done over multiple iterations until a structured rotated factor matrix was found and communalities of all remaining observed variables were greater than 0.20.

**Table 2:** Factor structure of the Moroccan version of Mini-MAC (24 items)

Items ^a^	Factors ^b^					h2	Item–total correlation	Alpha
	HH	AP	CA	FS	FA			
HH5	**.86**	−.13	−.12	.00	.05	.64	.74	.91
HH4	**.76**	.10	.02	−.02	.09	.65	.73	
HH7	**.71**	.08	−.01	−.11	−.05	.69	.78	
HH6	**.69**	.06	.12	.16	−.16	.56	.76	
HH2	**.62**	.16	.05	−.01	.00	.54	.70	
HH8	**.60**	.11	.02	−.15	−.12	.62	.83	
HH1	**.60**	.17	−.05	−.08	.02	.56	.76	
AP12	−.01	**.81**	−.01	.03	.02	.63	.78	.88
AP14	.03	**.80**	.03	.02	−.11	.71	.84	
AP11	.00	**.78**	−.08	−.02	.10	.59	.75	
AP16	.06	**.73**	.04	.01	−.04	.60	.78	
AP13	.05	**.65**	.05	−.03	−.01	.47	.68	
AP10	.22	**.49**	−.01	−.04	.09	.41	.63	
AP15	.25	**.37**	.02	.03	−.22	.42	.57	
CA24	.06	−.09	**.95**	−.02	−.04	.91	.91	.88
CA23	−.02	.06	**.89**	.04	.06	.91	.84	
CA21	−.08	.05	**.88**	.00	.03	.89	.87	
FS17	−.04	.02	−.02	**.91**	.02	.85	.91	.94
FS18	.00	.07	−.05	**.90**	−.01	.76	.84	
FS19	.03	−.09	.15	**.78**	.04	.77	.87	
FS20	−.26	−.17	.10	**.34**	.17	.51	.57	
FA26	.01	.05	.03	−.06	**.80**	.60	.70	.71
FA29	.01	−.11	.05	.19	**.70**	.25	.80	
FA28	−.28	.15	.07	.04	**.34**	.70	.50	
Eigenvalue	4.26	3.82	2.66	2.72	1.62			
Variance (total = 63%)	18%	16%	11%	11%	7%			

^a^Item number in the Moroccan Mini-MAC.

^b^Abbreviations for the original Mini-MAC subscales: *AP* Anxious Preoccupation; *HH* Helpless–Hopeless; *FS* Fighting Spirit; *FA* Fatalism; *CA* Cognitive Avoidance.

Accordingly, Items 3, 9, 22, and 27 cross-loaded significantly onto two different factors and were thus dropped from this model. Item 25 was removed because it failed to load significantly onto any factor. Despite the loss of five items in the factorial composition, the refined model replicated the five factors structure of the original version of Mini-MAC subscales (Helpless/Hopeless, Anxious Preoccupation, Fighting Spirit, Cognitive Avoidance, and Fatalism). The current five factors were constituted of the items making up the original scales. Hence, the factor names were maintained, and the five-factor model (model 1: HH, AP, FS, CA FA) was then assessed by CFA. The five factors, with eigenvalues between 1.62 and 4.26, and composed of 3 to 7 items, explained a total variance of 63% (Table 2).

### Internal consistency

The reliability of the Mini-MAC scale was assessed based on its internal consistency by determining Cronbach’s alpha coefficient. Cronbach’s alpha and item-total correlations (corrected) were calculated for each construct and statement item, respectively (Table 2). The Fatalism construct showed a minimum alpha value of 0.71, the remaining subscales exhibited alpha values between 0.88 and 0.94, which confirmed a very good internal consistency. The alpha values need to be at least 0.70 and ideally above 0.80 to be considered as a good consistency. This means that all constructs were reliable.

The minimum item-total correlation calculated was 0.50. The threshold for item-total correlations should be greater than 0.30 [41]. Table 2 illustrates that all of the constructs and statement items were unidimensional and had sufficient and acceptable internal consistency.

### Confirmatory factor analysis

#### Interscale correlations

The highest and most significant correlations (*p* < 0.001) were observed within two groups of factors, termed maladjustment (HH and AP) and positive adjustment (FS, CA, and FA) factors. Factors of the same group, either adjustment or maladjustment, correlated positively with each other. However, the correlations between the factors of the two groups correlated negatively (*r* = − 0.03 to − 0.70). FS, CA, and FA correlated negatively with HH and AP (*r* = − 0.03 to − 0.65) but correlated positively with each other (*r* = 0.43–0.77). AP showed a moderate negative correlation with Fatalism (*r* = − 0.42, *p* < 0.001) and Fighting Spirit (*r* = − 0.40, *p* < 0.001), and insignificant correlation with CA (*r* = − 0.03, *p* = ns). FS showed a high and positive correlation with Fatalism (*r* = 0.77, *p* < 0.01), and a moderate and positive correlation (*r* = 0.43) with CA. On the other hand, CA and Fatalism have a positive significant correlation (*r* = 0.56) (Table 3).

**Table 3:** Composite reliability, average variance extracted, maximum and average shared variance, and correlations between constructs

Latent Constructs	CR	AVE	MSV	ASV	Latent Constructs				
					1	2	3	4	5
1. Helpless-Hopeless	.95	.75	.62	.41	**.86**				
2. Anxious Preoccupation	.93	.66	.62	.24	.79^a^	**.81**			
3. Fighting Spirit	.97	.89	.59	.41	-.70^a^	-.40^b^	**.94**		
4. Cognitive Avoidance	.97	.93	.41	.20	-.30^b^	−.03	.43^b^	**.96**	
5. Fatalism	.93	.82	.59	.38	-.65^a^	-.42^b^	.77^a^	.56^b^	**.90**

b *p* < 0.01; a *p* < 0.001.

*CR* Composite reliability; *AVE* the square root of the average variance extracted; *MSV* Maximum Shared Variance; *ASV* Average Shared Variance.

#### Convergent validity

**Fig. 1:**
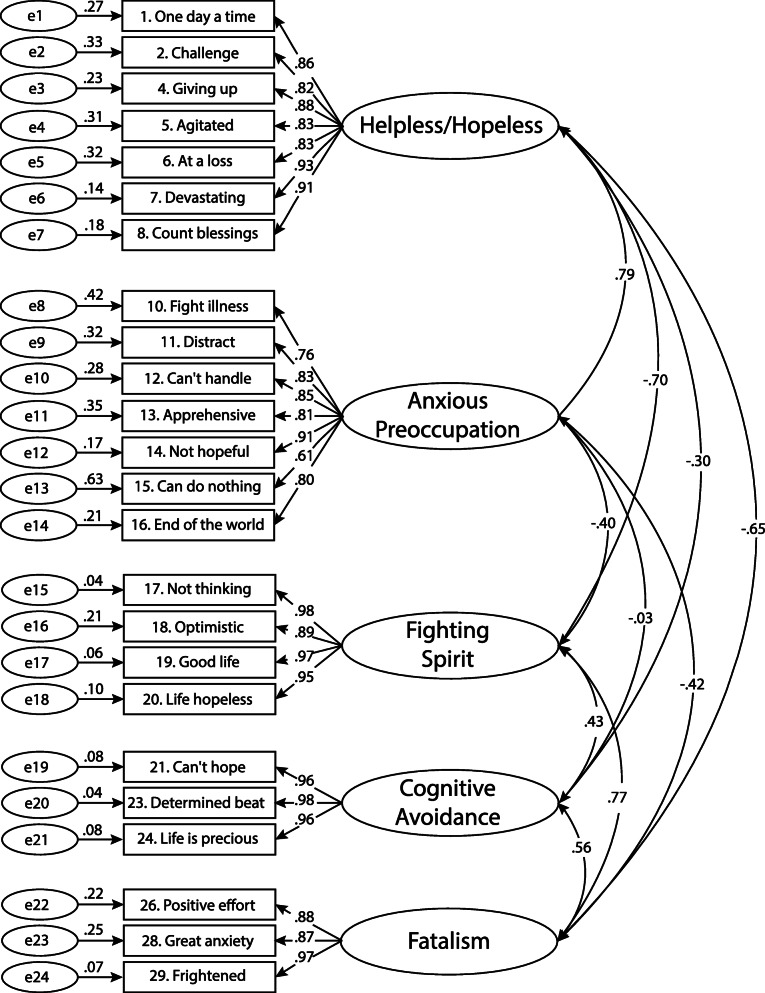
CFA measurement model

The first-order confirmatory factor analysis results also showed that the standardized regression coefficients exceeded 0.60; the smallest factors loadings (0.61) occurred at AP factor (item AP15). The remaining 23 factors were all greater than 0.70. In addition, the t-ratio (the t-value is calculated by dividing the parameter estimate by the standard error) associated with each factor-factor pair and factor-variable pair exceeded 1.96, which indicated a significant relationship with a *p*-value less than 0.05. The regression coefficients greater than 0.50 and the significant relationships associated with the high t-scores indicated that the first-order confirmatory factor analysis had statistically gathered acceptable evidence for convergent validity [41] (Fig. 1).

As the result of the CFA (Fig. 1), the structure of the hypothesized model of the 24-item Mini-MAC instrument was confirmed. The reliability and convergent validity of the instrument were also asserted, with high values for the CR (0.93–0.97) and AVE (0.66–0.93), respectively. Therefore, the whole process of factor analysis was confirmed, and the Mini-MAC instrument fitted the data fairly well (Table 3).

#### Discriminant validity, Fornell and Larcker criterion

In Table 3, the bolded values are the square root of Average Variance Extracted (AVE) of each dimension, whereas other values are inter-correlation among the latent factor dimension. The highest correlation value between factors was 0.79 (between HH and AP), while the smallest value among the square root of AVE values was 0.81. The findings warranted the discriminant validity of all model factors since the matrix diagonal values were higher than the off-diagonal values in the corresponding rows and columns. Average Shared Squared Variance (ASV) and Maximum Shared Squared Variance (MSV) were less than Average Variance extracted (AVE). HTMT value that is lesser than .85 or .90 [47], indicates a good discriminant validity. It appears from Table 4 that all matrix values are below 0.85, which pleads in favor of possible discriminant validity between all constructs of the proposed model. Overall, reliability and both convergent and discriminant validity tests indicated that the proposed constructs of the measurement model were justified at least with these two types of tests (Fornell and Larcker Criterion, and HTMT).

**Table 4:** Discriminant validity analyses: Heterotrait-Monotrait (HTMT) Criterion results

	Helpless-Hopeless	Anxious Preoccupation	Fighting Spirit	Cognitive Avoidance	Fatalism
Helpless-Hopeless	1				
Anxious Preoccupation	.76	1			
Fighting Spirit	.52	.30	1		
Cognitive Avoidance	.19	.06	.48	1	
Fatalism	.49	.29	.61	.35	1

#### Fitness of the measurement model

Evaluating the first-order measurement model included calculating the goodness-of-fit statistics and the standardized regression coefficients from the standardized model. The fit statistics for the first-order measurement model were χ^2^ = 225 (*p* = 0.77), root mean square error of approximation (RMSEA) =0.040, Goodness of Fit Index (GFI) = 0.99, adjusted goodness of-fit index (AGFI) = 0.98, and comparative fit index (CFI) =0.98, normed fit index (NFI) = 0.98, and Chi -square/ degrees of freedom (χ^2^/df) =0.93 (Table 5).

**Table 5:** Overall fit indices of the CFA model

	Absolute Fit			Incremental Fit			Parsimonious Fit
Fit index	χ^2^	RMSEA	GFI	AGFI	CFI	NFI	χ^2^/df
Observed Value	242 *p* = .77	.040	.99	.98	.98	.98	.93
Level of acceptance	*p* > .05	< .05	> .90	>.90	> .90	>.90	< 3

*RMSEA* root mean square error of approximation; *GFI* goodness of fit index; *AGFI* adjusted goodness of fit index; *CFI* Comparative fit index; *NFI* normed fit index;, *χ2* Chi-squared test*; df* Degrees of Freedom.

The fitness of the first-order model was assessed by three categories of fit statistics: absolute fit, parsimony correction, and incremental indices. These goodness-of-fit measures were highly acceptable when following the threshold values for fit statistics: the χ2/df should be less than 3, CFI should be greater than 0.95, NFI should be greater than 0.90, AGFI should be greater than 0.90, and the RMSEA should be less than 0.05 [41, 48]. Based on these ranges, all values were within acceptable threshold values. Therefore, the measurement model showed a good fit for the observed variables and relational contracting norm latent factors.

## Discussion

The major purpose of this work was to develop an Arabic version of the Mini-MAC instrument useful in the Moroccan context. To this end, two samples of 158 and 203 breast cancer women were investigated. The majority of women in the present sample were illiterate (67.5%) and housewives (65.9%). These quite specific sociodemographic characteristics are understandable in the light of the Moroccan reality where 52.6% of women aged 15 years old and above are illiterate, and only 24.7% are active [49]. The demographics of our sample are similar to those of several studies carried out in Morocco on patients with breast cancer [50–53], but certainly different from the characteristics of the samples used to validate the Mini-Mac in countries that differ from Morocco on a socio-cultural and economic level [27, 54].

Using exploratory and confirmatory factor analysis, and after dispatching five items, the current version was validated yielding the five-factors structure of the original version of the Mini-MAC instrument (Helpless/Hopeless, Anxious Preoccupation, Fighting Spirit, Avoidance, and Fatalism). The same structure, proposed originally by Watson et al., has been already validated by many authors working on different types of cancer [21–25, 27]. Our validated version showed similar psychometric properties as the original version [21], and other Mini-MAC validated versions [23, 25, 28–30].

The only difference between previous findings and ours resides in the degree of factors reliability and the factor intercorrelations. In this study, the coefficients reliability for AP, FS, CA, and FA are significantly high compared to some previous studies [21, 23, 24, 30], but seem nearly similar to others [25, 31].

The factors belonging to the group of passive coping strategies or maladjustment (HH/AP) are positively correlated with each other, but negatively correlated to those belonging to the group of active coping strategies or adjustment (FS, CA, and FA), and vice versa. This contradicted the original study [21] and corroborated that of Patoo [25], which demonstrated the same trend of correlation between these factors. CA showed a significant positive correlation with FS as well as with FA. Only the second association was pointed out in the original study [21], while the same results were reported by other studies [23, 24, 27, 30]. In line with Watson et al’s work [21], our findings also showed mild negative correlations between CA and AP, and a high negative correlation between CA and HH. The latter correlation was reported only by Patoo [25] who showed a mild negative correlation between the two factors. Indeed, these results, and in contrast to most previous studies, suggest that CA is an indicator of positive adjustment, and is positively associated with FS and FA. Nevertheless, many authors consider CA as an active distraction strategy that may facilitate problem-focused coping [25, 27, 32].

Some studies have grouped Mini-MAC factor adjustment to cancer into two types of strategies: passive coping strategies (Fatalism, Anxious Preoccupation, and Helpless/Hopeless), and active strategies (Fighting Spirit and Cognitive Avoidance) [22, 55]. However, this is not the case for the current study, where fatalism and Cognitive Avoidance were found as positive coping styles [25, 27], and not maladjustment as is stated by some authors [24, 28]. Besides, a Chinese study has divided Mini-MAC subscales into two groups called Negative and Positive Emotions. The first one, an indicator of maladjustment, includes Anxious Preoccupation and Hopelessness, whereas the second, indicator of positive adjustment, includes Fatalism and Fighting Spirit. The positive emotion group was found significantly associated with Cognitive Avoidance [27].

Fatalism showed a negative correlation with the factors of the passive coping strategies and a positive correlation with those of the active strategies. These findings corroborated some studies [24, 25, 30, 32], and at the same time contradicted others [22, 23, 28].

In contrast to western countries, Fatalism is considered as a positive coping strategy in Moroccan culture, the same results have been found in Persian, Korean, and Chinese countries [25, 27, 30]. Of particular note, Moslem people found their faith in destiny and fatalism, which has a different connotation from western countries; fatalism means acceptance and satisfaction based on the person’s reasons first, and then trust in God. This means that a positive attitude gathers fatalism with a fighting spirit. These findings are consistent with those of Islamic [25] and Asiatic versions [27, 30, 32] while they contrast with those of most western countries [22–24, 28]. They have asserted that Fatalism represents a positive adaptation and psychological battle with cancer. Our findings confirm the previous argument that FA is an adaptive coping tendency that does not correlate with distress [30, 31].

These interpretations should consider some caveats concerning methodological issues. In this light, most previous studies of Mini-MAC validation are based on exploratory factor analysis, which makes them less reliable [21, 23, 25, 27–31]. Whereas few studies are grounded on confirmatory factor analysis [22, 23, 32]. Additionally, the factor structure reported by different unstandardized studies should be variant due to many variables such as the type of cancer, sample size, culture, age, gender, patients, and phases of cancer. To deduce well-grounded conclusions about the reliability and validity of the Mini-MAC instrument for its future application, both analyses were adopted in the current study. The five factor measurement model showed an excellent fit according to the cut-off values of absolute, incremental and parsimonious fit indices. The fitness indices were supported by strong literature being referred [41]. The evidences of the validity and reliability aspects were infered from CFA measurements. However, this validity remains highly limited by place, time, and use of the scores resulting from the measurement operation, which was conducted on studies with small samples. Therefore, a study carried out at one location with one type of population may generate findings that are hardly to generalize to another sample from different location and/or population.

## Conclusions

Overall, this work represents the first validation of the Arabic version of the Mini-MAC instrument. We investigated its psychometric properties among a sample of 203 Moroccan breast cancer women using CFA to examine its factor structure. However, this study has some limitations that should be highlighted. The sampling was conducted in a single regional hospital and targeted a small sample size of cancer patients with a specific type of cancer. In addition, the sample is small and contains patients with different age range, cancer stages of the disease, and subject to different medical care at one Hospital. Hence, these findings cannot, however, be extrapolated to all Moroccan cancer patients from different regions. Thus, further studies on larger samples of different populations that depict diverse types, and stages of cancer are required. Moreover, longitudinal surveys are needed to assess the predictive validity of the scale for psychosocial outcomes. This short version of 24 items is a quick, valid, and reliable instrument in assessing cancer-specific coping of the adjustment response to cancer. It will allow physicians to know how negative and positive psychological adjustment to the illness could affect clinical practice. Hence, the acceptable psychometric properties obtained in this Arabic Mini-MAC guarantee its future use in clinical practice to measure various coping responses of breast cancer women.

## Data Availability

The datasets used and analyzed during the current study are available from the corresponding author on reasonable request.

## Abbreviations

AGFI: Adjusted goodness-of-fit index
AP: Anxious preoccupation
ASV: Average shared variance
AVE: Square root of the average variance extracted
χ^2^/ df: Chi square value/degrees of freedom
CA: Cognitive avoidance
CFA: Confirmatory factor analysis
CFI: Comparative fit index
CFI: Bentler comparative fit index
CR: Composite reliability
EFA: Exploratory factor analysis
FA: Fatalism
FS: Fighting spirit
GFI: Goodness of fit index
HH: Helpless/hopeless
HTMT: Heterotrait-monotrait criterion
Mini-MAC: Mini mental adjustment to cancer
MSV: Maximum shared variance
NFI: Normed fit index
RMSEA: Root mean square error of approximation
